# Failure probability assessment of landslides triggered by earthquakes and rainfall: a case study in Yadong County, Tibet, China

**DOI:** 10.1038/s41598-020-73727-4

**Published:** 2020-10-05

**Authors:** Lixia Chen, Le Mei, Bin Zeng, Kunlong Yin, Dhruba Pikha Shrestha, Juan Du

**Affiliations:** 1 Institute of Geophysics and Geomatics, China University of Geosciences, Wuhan, 430074 China; 2 Environment Faculty, China University of Geosciences, Wuhan, 430074 China; 3 Engineering Faculty, China University of Geosciences, Wuhan, 430074 China; 4 Department of Earth Systems Analysis, Faculty of Geo-Information Science and Earth Observation (ITC), University of Twente, Enschede, 7500 AE The Netherlands; 5 Three Gorges Research Centre for Geohazards, China University of Geosciences, Wuhan, 430074 China

**Keywords:** Environmental sciences, Hydrology, Natural hazards

## Abstract

Yadong County located in the southern Himalayan mountains in Tibet, China, is an import frontier county. It was affected by landslides after the 2011 Sikkim earthquake (Mw = 6.8) and the 2015 Gorkha earthquake (Mw = 7.8). Casualties and property damage were caused by shallow landslides during subsequent rainfall on the earthquake-destabilized slopes. Existing researches have generally examined rainfall- and earthquake-triggered landslides independently, whereas few studies have considered the combined effects of both. Furthermore, there is no previous study reported on landslide hazards in the study area, although the area is strategically applicable for trade as it is close to Bhutan and India. This study developed a new approach that coupled the Newmark method with the hydrological model based on geomorphological, geological, geotechnical, seismological and rainfall data. A rainfall threshold distribution map was generated, indicating that the southeast part of Yadong is prone to rainfall-induced landslides, especially when daily rainfall is higher than 45 mm/day. Permanent displacement predictions were used to identify landslide hazard zones. The regression model used to calculate these permanent displacement values was 71% accurate. Finally, landslide probability distribution maps were generated separately for dry and wet conditions with rainfall of varying intensities. Results can serve as a basis for local governments to manage seismic landslide risks during rainy seasons.

## Introduction

Earthquakes are an important triggering factor for the mass movement processes; however rainfall is another crucial factor in any slope failures. After landmass experiences a powerful earthquake, the periods of excessive rain can render sloping areas very vulnerable to landslides, as the groundwater rises and the shear strength of the soil matrix declines. Existing studies have created independent rainfall- and earthquake-triggered landslide probability assessments based on local environments^1–7^; however, they have largely ignored their interactions. Therefore, we must consider both these two triggering factors to understand landslide occurrences and determine hazard zones for areas that are subjected to both.

For earthquake-induced landslides, deformation and failure probabilities have been assessed using statistical analysis^8–14^ and Newmark modelling^15^ integrated with Geographic Information System (GIS) techniques^16–20^. The Newmark method, along with its modified forms, is the most widely used technique for region-scale seismic landslide hazard assessments because it is a simple tool for calculating co-seismic displacement. Jibson et al.^21^ applied a simplified Newmark method to analyse seismic landslide deformation based on the data from historical earthquakes and landslides in Northridge, California. Their research showed that regional topography, geotechnical parameters, and seismic intensity could be used to calculate the failure probability of a slope during a seismic event. Romeo^22^ incorporated peak ground acceleration and Arias intensity during an earthquake into the Newmark method and assessed landslide deformation based on 189 seismic acceleration digital records from 17 earthquake events in Italy. Beyabanaki et al.^23^ discovered the failure behaviour of a landslide triggered by the Wenchuan earthquake by applying disk-based discontinuous deformation analysis. These results provide a useful reference for understanding the mechanism of earthquake-induced landslides. When researchers lacked important information, such as historical landslide inventories or input parameter values, they have proposed simple approaches that yield acceptable results, such as using statistical patterns to estimate the missing data^24,25^.

For rainfall-induced landslides, failure probability is typically assessed using statistical models^2,26–29^ or using deterministic models^30,31^. In a data-poor environment, researchers must choose the right method and maximise its usability with whatever field data is available. When long term data on rainfall and landslide occurrence are available, landslide probability can be determined in a statistical way, such as analysing the regional relationship between landslide occurrences and rainfall patterns or intensities. When the data on rainfall and landslide occurrences and rainfall is limited, deterministic models can be used. One of the earliest deterministic models in this field combined a hydrological model with an infinite-slope model to study the different hazard levels of rainfall-induced landslides using GIS techniques^31^. Subsequent studies have generally followed the approach of combining a hydrological model with a physical slope model to simulate the instability of rainfall-induced slope movements^32–34^. Statistical methods require the historical records of landslide occurrences and long-term rainfall data, which may not be available everywhere, especially in developing countries. In the cases of data scarcity, statistical methods are unsuitable for assessing landslide failure probability; therefore, combining hydrology and slope stability models has proven to be a good solution to the problem.

That said, earthquake-prone areas that also experience periods of heavy rainfall require a way to simultaneously address the potential movement of unstable slopes triggered by earthquakes and precipitation during the subsequent rainy season. In the past decade, researchers have attempted to simultaneously explore both of these effects. They have used a range of approaches from developing GIS-based neural networks^35^ to using the groundwater depth as an alternative for the slope failure zone^36^ and then to comparing multiple models in terms of the prediction accuracies^19^. However, focused efforts are required for the realistic characterisation of slope groundwater conditions and material properties^37,38^. This recent research has blended databases or compared susceptibility models to assess slope failure probability (due to rainfall and earthquakes). It has not considered the sequential relationship of these two factors, nor has it identified the essential mechanisms by which post-earthquake rainfall triggers landslides.

Yadong County is located in the southern part of the Tibet autonomous region, near the China–India, and China–Bhutan borders. It is part of an important trade corridor between China, India, and Bhutan; therefore, rapid economic growth and infrastructure development are anticipated for this area. However, Yadong is located in the tectonically active Qinghai–Tibet Plateau, where 43% of all Eurasian earthquakes above magnitude 7 are centred^10^. Two powerful earthquakes have damaged Yadong recently, inflicting severe economic losses and human casualties. The first was the Sikkim earthquake (Mw = 6.8), which struck just over the Indian border on 18 September 2011 (https://news.now.com/home/international/player?newsId=12990&refer=), killing seven people and injuring 22. The area was affected again by the Gorkha earthquake (Mw = 7.8) in Nepal, which occurred on April 25, 2015. The epicentre of the Sikkim earthquake was approximately 40 km from Yadong, whereas that of the Gorkha earthquake was 380 km away; hence, despite its weaker magnitude, the Sikkim quake caused more damage in Yadong. The Gorkha earthquake was followed by a serious of aftershocks of magnitude Mw > 6, including a powerful event (Mw = 7.3), which occurred on May 12, 2015^39^.

The Sikkim and Gorkha earthquakes (and their aftershocks) triggered many landslides, and the rainfall events that followed the earthquakes induced more potentially unstable slopes. Compared with the impacts in other Chinese frontier counties, the landslides in Yadong triggered by these two earthquakes were not particularly serious. However, because of its important economic links to India and Bhutan, Yadong faces a high economic risk due to landslides. Therefore, it is essential for local citizens and governments to track landslides and to assess their risks during an earthquake and subsequent rainfall events. Unfortunately, only a few detailed maps of Tibetan landslide inventories, geospatial databases, and some climate data exist for assessing landslide failure probabilities.

The main objective of this study is to assess the landslide failure probabilities in the data-poor environment, while simultaneously considering two triggering factors: earthquakes and rainfall. We coupled a hydrological model with the Newmark method to assess earthquake-induced landslide failure probabilities. Several essential maps were generated e.g. factor of safety (FOS), rainfall threshold, permanent displacement and landslide failure probability maps. These tools are intended to help the residents, governments and emergency managers to quickly assess the threats that landslides pose to lives and infrastructure. We hope that our methodology could be useful in assessing landslide failure probability in similar regions with scarce data.

## Study area

### Geography and climate

**Figure 1:**
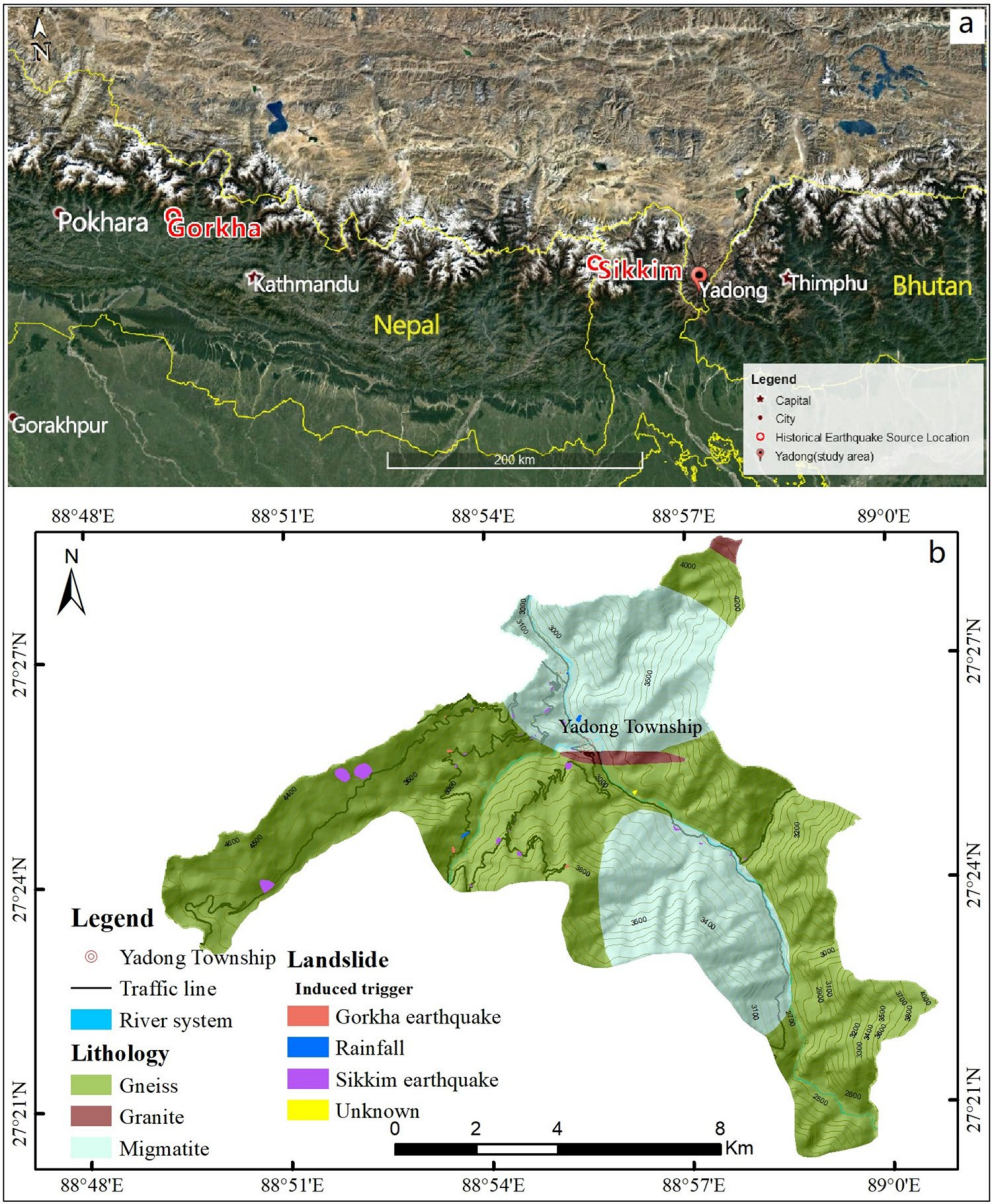
The study area is located in Yadong County in southern Tibet, China. (**a**) The Google Earth image fragment (https://www.google.com/earth/) of the area also shows the locations of the Gorkha and Sikkim earthquakes. (**b**) The lithology map of Yadong County, prepared by Le Mei using ArcGIS ver. 10.2 (https://www.esri.com/), is based on the geological map (1:250,000) downloaded from the China Geological Survey (https://www.ngac.org.cn/Data/FileList.aspx?MetaId=F5F68B349ADE1B60E0430100007F0760&Mdidnt=d00121956).

Yadong County, which lies within the geographic coordinates 88° 49′ 15″–89° 0′ 49″ E and 27° 20′ 14″–27° 28′ 34″ N, is located in the middle of the Himalayan mountain range in southern Tibet, China, bordering India and Bhutan (Fig. 1). It covers a surface area of approximately 95 km^2^, with elevation varying from 2330 to 4730 m a.s.l. The county comprises two administrative townships (Xiasima and Xiayadong). The Yadong river and its tributaries form the main hydrological system in the area. It flows across the centre of the county to the south before entering Bhutan. The southern part of the county is dominated by high mountainous terrain (elevation of 3460–4730 m) that is cut by steep valleys.

The land use in the study area is mainly forest and grassland, which together cover roughly 80% of the county. The settlements are mostly clustered around the Yadong river, covering a surficial area of 0.18%. They are densely populated where development is possible (Fig. S1).

Annual precipitation varies from 1100 to 1500 mm, almost all of which (98%) falls during the rainy season (April to October). The highest monthly average precipitation is 244 mm, which occurs in July (Fig. S2). The area also has vertical climatic zones because of the significant variations in elevation. Below 2700 m elevation the climate is humid subtropical where a large majority of the population and human activities are concentrated. The area receives more rain than that in other Tibetan counties. For instance, the weather station in the nearby Sa'gya county (88° 00′ E, 28° 54′ N) recorded annual rainfall of just 339 mm in 2011.

### Geology

The rock units in Yadong are mainly Precambrian and Quaternary according to the geological map (scale 1:250,000) and report provided by the China Geological Survey. The Precambrian formations cover 94% of the total area and comprise granite, gneiss, and migmatite. These formations have been highly fractured by the Himalayan orogeny; hence, they are more susceptible to weathering, and thus dominate the shallow landslides. The foot slopes are covered by Quaternary alluvium and talus.

Tectonically, the study area is distributed in the middle of the Himalayas orogenic belt. Ductile deformation and nappe structures are well developed, which causes two groups of structures: E- and NNE-oriented faults. The current, widely distributed geothermal in Yadong indicates that most faults are currently in an active state.

## Results and discussion

### Inventory of landslides and soil depth simulation

The landslides were probably triggered by earthquakes or by rainfall following the earthquakes. In Table S1 an inventory of the landslides identified during the fieldwork and their detailed information are given. These landslides were probably induced by the 2011 Sikkim earthquake, or the 2015 Gorkha earthquake, or by rainfall during the rainy season. The two earthquakes triggered in total 38 landslides in Yadong (32 by the Sikkim earthquake; 6 by the Gorkha earthquake and its aftershocks). Six additional landslides were initiated during rainy days following the Sikkim earthquake.

Most of these landslides were debris slides according to their classification by Varnes (1978)^40^. The landslide body predominantly consists of coarse-grained soil with a depth measuring less than 8 m. Detailed information, such as landslide magnitude, lithology of bedrock, and type of movement, was shown in Figs. 1, S3, Table S2. Most landslides were originated from gneiss bedrock and were caused by the Sikkim earthquake. The earthquake loosens the rock structures due to shaking, which decreases the rock strength. Rainfall following the earthquakes worsens the stability of slopes because of water infiltration. The depth of the slope movements is predominantly shallow, with the average value 3 to 5 m. For the following prediction of landslide spatial distribution, soil thickness in the whole area was interpolated with UK method and found to be less than 12 m (Fig. S4).

### The factor of safety evaluation

**Figure 2:**
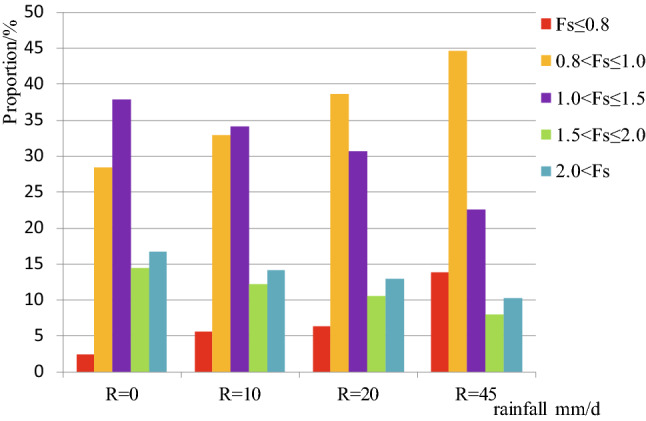
Histograms showing the area percentage with different safety factor values for the study area under four daily rainfall categories, *R* (Eq. (4)): 0 mm/day, 10 mm/day, 20 mm/day and 45 mm/day. When daily rainfall increases potentially vulnerable areas also increase.

The study shows that nearly 16% of the study area under dry conditions has very low FOS values (below 0.8), suggesting that many slopes are potentially vulnerable to landslides, even under normal conditions without rain (Fig. S6a). During the rainy season, an increase of daily rain amount means the increase in the vulnerable area (Fig. 2). When daily rain is more than 45 mm, more than half of the area seems to be potentially vulnerable (Fig. S6).

### Determination of daily threshold rainfall

**Figure 3:**
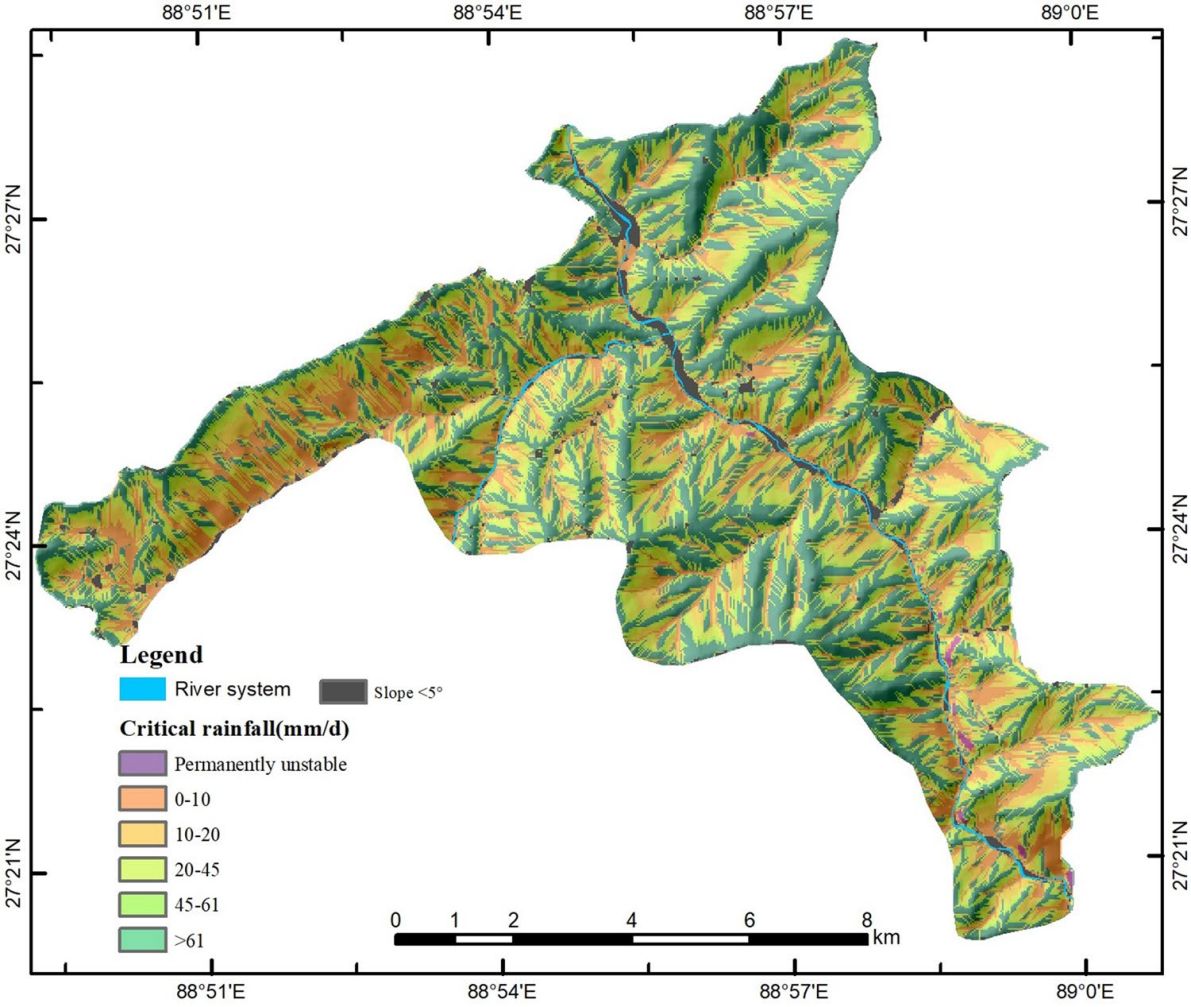
Map of the spatial variation of rainfall threshold for landslide hazard. This map was generated using Eq. (5) by Le Mei and Lixia Chen on ArcGIS ver. 10.2 (https://www.esri.com/).

The result of the daily rainfall threshold map provides the minimum rainfall value, which is necessary to trigger slope movement for each grid cell (25 m × 25 m) in the area (Fig. 3). Some areas may show unstable tendency even when daily rain is minimal. The percentage of the study area with each rainfall threshold interval is presented in Table S3.

The study shows that the unconditionally unstable category contains cells with FOS values that are lower than 1.0 even under dry conditions. This category covers 8% of the total study area and is primarily concentrated in the south-eastern part, where the terrain is particularly steep. The study carried out in a similar environment in the high and very high mountain region in Nepal shows the possibility of rock and debris slide hazard above 4000 m due to freeze–thaw action, which occurs mainly during the summer months with air temperature fluctuating around the freezing point^41^. The study shows that nearly 37% of the area in the Yadong county requires a very high amount of rain (more than 60 mm a day) to trigger landslides. Looking at the climatic data, this situation rarely seems to occur. This category tends to include areas at higher elevations or gentler slopes. The area with a daily rainfall below 20 mm accounts for 28% of the total area. This category dominates the area where slopes are steeper than 32 degrees; however, most of these slopes are located in the uninhabited portions of the valley.

The study based on the historical rainfall-induced landslides suggests that they were triggered by a large amount of rainfall (Table S3). Approximately 22% of landslides were triggered by daily rainfall lower than mm. The rest were mostly triggered by daily rainfall higher than 20 mm, which is much higher than the triggering rainfall intensities in other Tibetan counties.

### Validation of landslide hazard during the 2011 Sikkim earthquake

The number of landslide grid cells in each class of permanent displacement was calculated and compared for the three regression models (Table 1). The results obtained by using Eq. (7) shows that the number of landslide cells in the very high and high classes of permanent displacement is nearly twice as large as those using Eq. (6). On the other hand, the results obtained by using Eq. (8) categorised less than 1% of cells as very high or high, the area of which does not capture most of the historical landslides, even though this equation was developed in southwest China.

**Table 1:** Comparison and validation of permanent displacement calculated by three regression models, using records of landslides and earthquake intensity during the Sikkim earthquake.

Regression models	Number of landslide grids in each class of permanent displacement				
	Very low	Low	Moderate	High	Very high
Equation (6)^21^	27	152	152	120	0
Equation (7)^42^	8	35	169	231	8
Equation (8)^44^	287	102	58	4	0

The study shows the accuracy rate of permanent displacement values is 0.713 (Fig. S7), indicating that Eq. (7) is the best option for calculating permanent displacement in Yadong County. An accuracy of 0.713 is not extremely high; however, this still shows promise as a tool to support the local governments of southern Tibet as they seek to improve their landslide hazard risk management.

The regression model provided by Chousianitis et al.^42^ for permanent displacement calculations proved to be very suitable in the studied area, and the logarithm of this model is the best for evaluating permanent displacement in Yadong. The major advantage of this model is the parameter of critical acceleration in the formula.

There is still room to improve on the regression models for permanent displacement calculation, and they may need to be tailored for specific regions, such as for southern Tibet. Further development of these models is needed in the future. Future studies may choose to include other parameters, such as soil permeability, seismic intensity, or the effects of topography on seismic amplification, as a way of improving model performance. For instance, during major earthquake events, seismic waves generally transmit from the source to the ground surface, where topography can cause significant acceleration/amplification, resulting in earthquake-induced landslides. This needs to be further studied.

The result shows landslide permanent displacement and the corresponding failure probabilities, drawn from the analysis of the 2011 Sikkim earthquake (Table S4).

### Landslide failure probability estimation for the extreme earthquake scenario and different daily rainfalls

**Figure 4:**
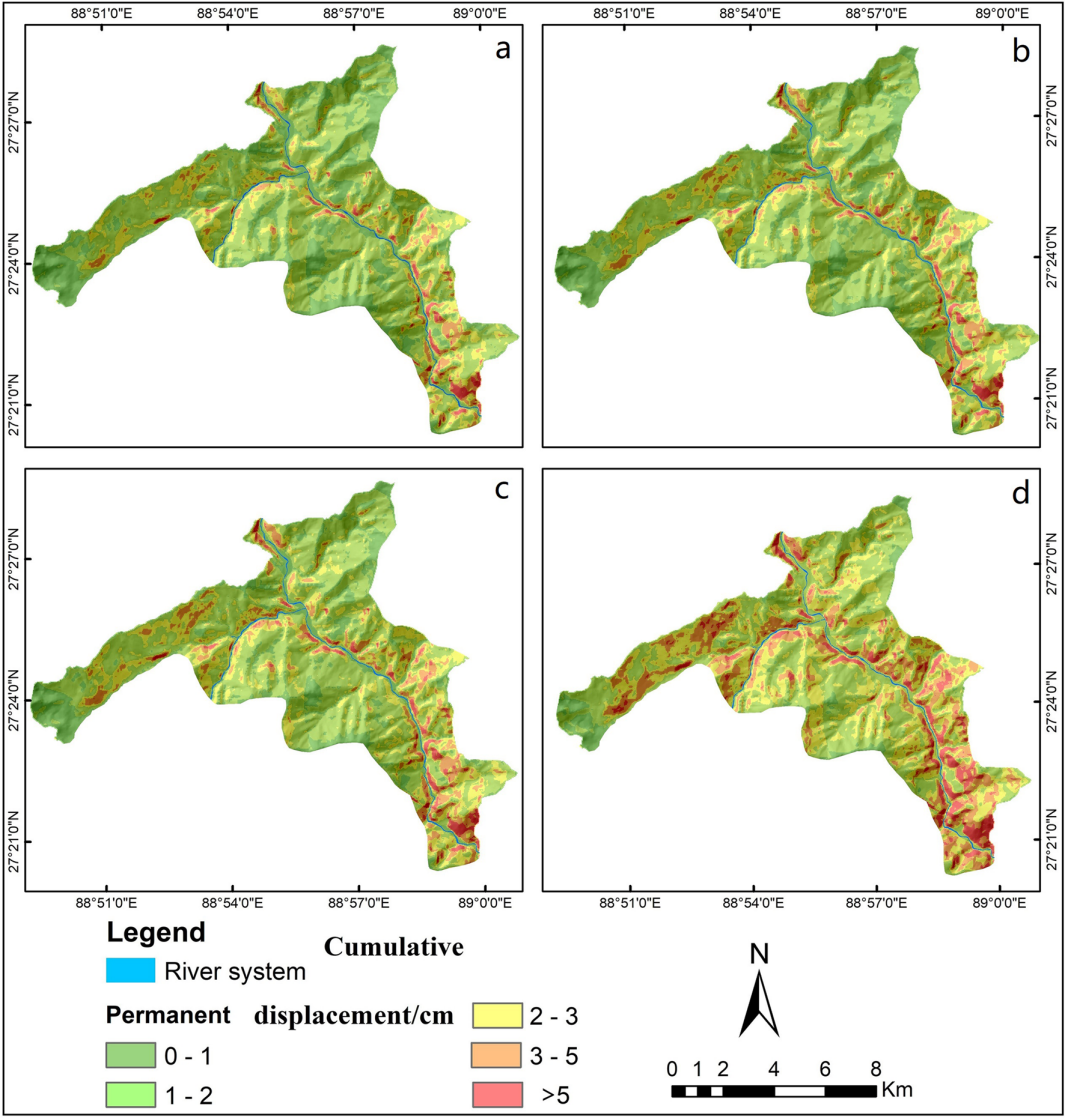
Permanent displacement distribution maps in the study area under (**a**) dry conditions and rainfall categories of (**b**) 10 mm/day, (**c**) 20 mm/day, and (**d**) 45 mm/day, when combined with a 10% probability of a ground motion exceeding 0.20 g in 50 years. These maps were generated by Le Mei and Lixia Chen using Eq. (7) on the ArcGIS ver.10.2.

**Figure 5:**
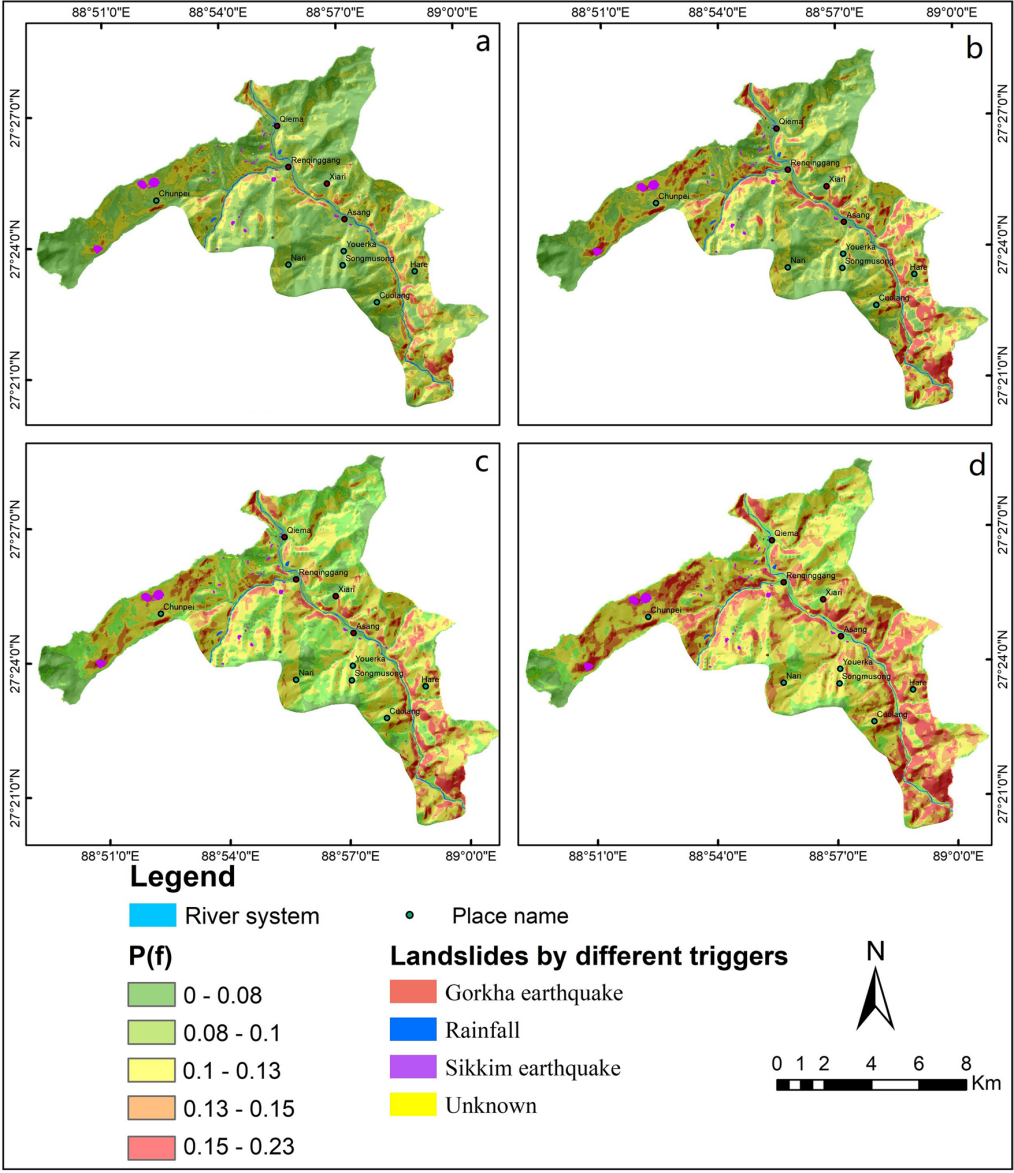
Landslide failure probability maps under (**a**) dry conditions and rainfall intensities of (**b**) 10 mm/day, (**c**) 20 mm/day, and (**d**) 45 mm/day, when combined with a 10% failure probability of a ground motion exceeding 0.20 g in 50 years. These maps were generated by Le Mei and Lixia Chen using Eq. (7) on the ArcGIS ver.10.2.

Maps (Figs. 4, 5) of landslide permanent displacement and failure probability corresponding to different daily rainfalls were generated for an earthquake scenario (10% probability of a ground motion exceeding 0.20 g in 50 years).

**Figure 6:**
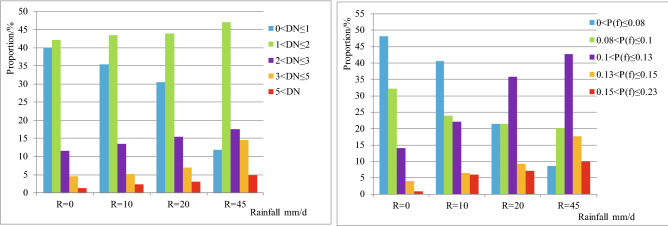
Area percentages of (**a**) landslide permanent displacement and (**b**) failure probability in the study area for different daily rainfall, *R* (Eq. (4)), of 0 mm/day, 10 mm/day, 20 mm/day and 45 mm/day.

This study shows that rainfall had a significant influence on the results of landslide permanent displacement. As daily rain increases, the proportion of high-hazard areas also increases dramatically, while there is a significant decrease in the proportion of low-hazard areas (Fig. 6). Under dry conditions (*R* = 0 mm/day), the area with a *D*_*N*_ value of more than 5 cm accounts for 0.88%, and a *P(f)* above 0.15 accounts for 0.84%. During a reasonable day during the rainy season (with daily rainfall of 10 mm), the area percentage with very high landslide failure probability would be about 7 times higher than that in dry conditions. Moreover, under intense rainfall (*R* = 45 mm/day), the area percentages increases to 6% and 11%, respectively. The results clearly show that the seismic landslide risk posed by any given earthquake will be strongly dependent on rainfall.

## Conclusions

The study aims to assess the regional failure probability of seismic landslides under different rainfall scenarios in Yadong, Tibet, where hazard and rainfall data are currently limited and risk assessment is urgent because of strategically important trade between China, Bhutan, and India. The presented method combined the Newmark model and the hydrological model to determine landslide safety factors, permanent displacement, and failure probability metrics for different scenarios. This allowed us to predict landslide occurrence in response to earthquakes and daily rainfall. We demonstrated that the hydrological effect is important in determining seismic landslides in the rainy season. The study shows that the method is capable of identifying critical areas for hazard risk control and management. The method also functions as a tool to find the threshold rainfall for areas where landslides or unstable slopes exist, and where rainfall is concentrated.

This method relies on data or estimates of slope gradient, soil depth, geotechnical parameters, seismic intensity and daily rainfall data. Slope gradient can be generated easily using freely available digital elevation data and data on daily rainfall is widely available in most places around the globe. With minimal field sampling of representative soils, this method can be applied in other regions with minimal data. We hope that this method may serve as a basis for hazard and risk control in mountainous areas where landslides are triggered by earthquakes just before or during the rainy seasons.

## Methodology and data

**Figure 7:**
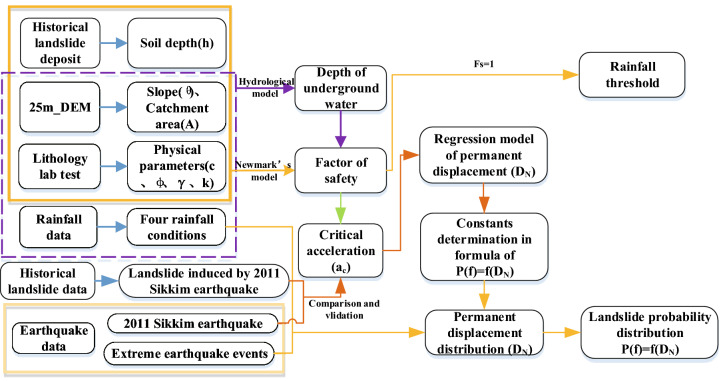
Flowchart of seismic landslide hazard probability assessment using the combination of the hydrological and infinite slope model in the Newmark method.

To conduct this regional study, we selected the hydrological model to determine groundwater depth. Based on the margin of safety factor, a rainfall threshold distribution map was then produced, which was used as an input for the Newmark model which has previously been successfully implemented to predict the locations of shallow, unstable slopes induced by earthquakes^43^. We tested the ability of three regression methods (by Jibson et al.^21^, Chousianitis et al.^42^, and Jin et al.^44^) to calculate the permanent displacement of landslides using the hazard data during the 2011 Sikkim earthquake. The model with a highest accuracy by ROC curve^45,46^ was chosen to predict landslide permanent displacement and failure probability considering rainfall conditions and future extreme earthquake intensities. The flow chart of our methodology is presented in Fig. 7.

### Critical acceleration calculation

We based our slope failure predictions on the Newmark method, which was first developed to estimate co-seismic slope displacement and later proved to be useful in estimating earthquake-induced slope displacement^11,21,47–50^. As is typical for the Newmark method, critical acceleration is defined based on the assumption that a landslide would experience no permanent displacement at any acceleration below the critical or yield level^20^. By treating a landslide as a rigid-plastic body, the critical acceleration is the acceleration that overcomes the frictional resistance, which initiates the sliding movement down the inclined plane. We used a simplified equation to calculate this critical acceleration, wherein it is a function of a landslide static FOS (factor of safety) and the landslide geometry as follows:

1 $$a_{c} \,=\, (Fs - 1)g \sin \alpha ,$$

where *a*_c_ is the critical acceleration; *g* is the acceleration caused by Earth’s gravity; *Fs* is the landslide FOS; and *α* is the potential sliding surface inclination angle, which is approximately equal to the slope angle based on grid cell calculation.

### The factor of safety calculation

The factor of safety is a measurement of slope stability, which is defined as the ratio of the net resisting force divided by the net driving force. If the value of the FOS is less than 1.0, then the slope is considered unstable. As shown in Eq. (1), the FOS is an important indirect input in the Newmark method. We used the infinite-slope model to calculate the FOS, assuming that the slope is under stress as a uniform block sliding over an infinite inclined surface^51^. This static FOS is expressed as follows:

2 $$Fs = \frac{c}{\gamma d\sin \alpha } + \frac{\tan \varphi }{{\tan \alpha }} - \frac{{m\gamma_{w} \tan \varphi }}{\gamma \tan \alpha },$$

where *c* is the effective cohesion (KPa); *φ* is the effective internal friction angle; *γ* is the unit weight of the slope’s soil (KN/m^3^); *d* is the potential landslide thickness (m), which is deeply described in the section of Data sources; *α* is the potential sliding surface inclination angle; *m* is the ratio of groundwater depth, *h* (m), to potential landslide thickness, *d*; and *γ*_*w*_ is the unit weight of water (KN/m^3^).

### Determination of groundwater depth and rainfall threshold

In Eq. (2), groundwater depth, *h*, is an important parameter, which we determined by using the hydrological model^52^. The hydrological model assumes that rainfall in the upstream catchment basin infiltrates into the soil, and that the permeability of the soil does not change with depth. Furthermore, run-off on the slope can reduce the amount of rainfall infiltration. The percentage primarily depends on the slope angle and soil permeability. However, in this area, the soil is relatively loose and the rocks have been vibrated and cracked by previous earthquakes. Top layers of soil and weathered rocks are generally highly permeable. The vertical infiltration is relatively high. Therefore, we assumed that all the rain infiltrates into the soil. According to Darcy's law, the discharge, *Q*, through a certain cross-section of the slope under rainfall can be expressed as follows:

3 $${\text{Q}}\, { = }\,hw\, \cos\,\alpha \cdot v$$

where *w* is the width of the slope (and for raster-based calculations is equal to the grid size); and *v* is the seepage velocity, defined as *v* = *ki*, where *k* is the permeability coefficient, which is determined by the physical properties of the slope; and *i* is the hydraulic gradient, where $i = \sin \alpha$. Owing to the landslide types (debris slide) in our case study area, the geological model for the slopes can be treated as an infinite slope model, which assumes that the groundwater table is parallel to the ground surface. Therefore, under a certain daily rainfall, *R*, the height of the groundwater table, *h*, was deduced as follows:

4 $$h = \frac{RA}{{kw\sin \alpha \cos \alpha }},$$

where *A* is the catchment area, which was determined by the flow of water through each cell of the grid by tracking water flow direction and estimating the cumulative amount. The catchment area was calculated using the hydrological analysis module of ArcGIS. To use this module, we subjected a digital elevation model (DEM) of the study area to a filling process, which eliminated any closed drainage basins. The water flow direction through each cell was then calculated based on the filled DEM. The catchment area at any given point was estimated by counting the number of contributing cells and multiplying this by the cell size.

After combining Eqs. (2) and (4), we determined the daily rainfall threshold, which is the minimum value of daily rainfall required to trigger a slope failure. Assuming that a landslide is triggered when the FOS is 1.0, the rainfall threshold, *R*_*t*_, can be expressed as follows:

5 $$R_{t}\, { = }\,\frac{kdw\cos \alpha }{A}\sin \alpha (\frac{\gamma }{{\gamma_{{\text{w}}} }})\left[ {\left( {1 - \frac{\tan \alpha }{{\tan \varphi }}} \right) + \frac{c}{{\gamma_{{\text{w}}} d\,{\cos}\,\alpha\, {\tan}\,\varphi }}} \right].$$

### Landslide permanent displacement calculation

The Newmark method does not evaluate slope instability based on the minimum value of the safety factor but instead uses the permanent displacement of the sliding mass along the slip surface. In response to seismic shaking, landslide displacement can be calculated by the multivariate regression method. The displacement of a landslide induced by an earthquake has a functional relationship with the critical acceleration (see Eq. (1)) and the Arias intensity.

The regression method allows for the calculation of the permanent displacement of slopes in terms of seismic intensity, morphological figures and the physical and mechanical parameters of the materials involved. For the landslides induced by the Sikkim earthquake in Yadong, we referred to the most suitable regression models among the previous studies^19,21,42,44^.

Jibson et al.^21^ used the critical acceleration, *a*_c_ and the Arias intensity, *I*_*a*_, to fit the regression equation based on the data from 280 recording stations during 13 earthquake events:

6 $$\log D_{N} { = }1.521\log I_{a} - 1.993a_{c} - 1.546,$$

where *D*_*N*_ is the permanent displacement of the landslide (cm); *I*_*a*_ is the Arias intensity (cm/s), an index for measuring seismic intensity that can be calculated from the peak ground acceleration (PGA) of the site; and *a*_c_ is the critical acceleration from Eq. (1). PGA is not a measure of the total energy of an earthquake, but rather how much the ground moves at a given geographic point.

Chousianitis et al.^42^ fitted different permanent displacement calculation formulas, using *I*_a_ values from 0.4 cm/s to 120.3 cm/s. Their formula is suitable for areas with relatively few data as follows:

7 $$\log D_{N} = 2.228\log I_{a} - 2.498\log a_{c} + 0.373\log I_{a} \log a_{c} - 5.495,$$

Jin et al.^44^ provided a modified Newmark model to assess the landslide hazard triggered by the Lushan earthquake in southwestern China as:

8 $$\log D_{N} = 0.215 + \log \left[ {\left( {1 - \frac{{0.7a_{c} }}{{a_{\max } }}} \right)^{2.341} \left( {\frac{{0.7a_{c} }}{{a_{\max } }}} \right)^{ - 1.438} } \right],$$

where *a*_*max*_ is the PGA.

We needed to determine the Arias intensity, *I*_a_, prior to using these equations. This parameter can be calculated as a function of PGA, distance to the epicentre, or seismic intensity^42,53^. Of these, Romeo^22^ used PGA value to calculate *I*_*a*_ on the basis of 190 measured seismic acceleration curves, and proposed the following equation:

9 $$I_{a} = 0.004PGA^{1.668} ,$$

where PGA is peak ground acceleration in cm/s^2^.

The accuracy of the permanent displacement calculation was tested using the Receiver Operating Characteristics (ROC) curve^45,46^, with the area under the ROC curve used to assess the success rate. The equation to calculate permanent displacement with the highest the success rate is selected among the Eqs. (6)–(8).

### Landslide failure probability calculation

It is possible to obtain a relationship between the *D*_*N*_ and the probability of landslide instability. Jibson et al.^21^ established a regression equation between landslide failure probability, *P(f)*, and *D*_*N*_ based on a large number of historical earthquake-induced landslide records:

10 $$p(f) = k[1 - {\exp}(a \times D_{N}^{b} )],$$

where the coefficient *k* represents the maximum value of landslide failure probability, and *a* and *b* are constants. The values of *k*, *a* and *b* were determined from the data of historical landslides and earthquake intensity during the Sikkim earthquake. During the simulation, the landslide area percentage in each range of permanent displacement was counted as the *P(f)*, the function of which was fitted in MATLAB (Mathworks, USA).

### Data sources

In the absence of historical data on landslide and climate, the data used in this study came from field investigations, laboratory tests and the application of the Newmark method and hydrological model.

#### Slope angle

The slope angle is essential in the calculation of landslide safety factor and critical acceleration (Eq. (2)). We derived a slope angle map (Fig. S5) from a 25 m resolution DEM, downloaded from the National Catalogue Service for Geographic Information System (https://www.webmap.cn/main.do?method=index).

#### Geotechnical parameters

For slope failure probability estimation, geotechnical parameters are required, the characterisation of which was difficult to characterise these parameters on a regional basis. On the basis of the field and laboratory tests of weathered soils from different bedrocks, we summarised the geotechnical parameters for each geologic unit (Table S5).

#### Soil depth

In this study, we assumed that the potential landslide thickness is the soil depth to the bedrock. Therefore, soil depth is another important input data that has an effect on landslide FOS calculations^54^. The majority of landslide constituents in the study area is soil from weathered rock, and the sliding surface is usually along the soil–bedrock interface. During field survey, the soil thickness of each exposed profile was measured using a rangefinder. Additional soil thickness data was available from some trenches and old landslides. In total, 149 discrete thickness value points distributed across the study area were measured.

Despite these efforts, soil thickness data still did not cover the entire study area. To compensate for this, we used universal kriging (UK) to convert the point data into continuous planar data.

#### Seismic intensity

We derived the expected PGA values for Yadong from zonation maps of the seismic ground motion parameter from the China Bureau of Earthquakes (Fig. S8). The PGA value for our study area was 0.2 g, which reflects a 10% probability of exceedance within 50 years.

#### Rainfall data

In Yadong precipitation records are only available after 1 May 2014. This greatly limited our prediction of rainfall scenarios through statistical method, such as frequency-magnitude analysis. Instead, we classified daily rainfall data into four intervals: 0–10 mm/day, 10–20 mm/day, 20–45 mm/day, and greater than 45 mm/day (Fig. S9), because these intervals can represent common situations in Yadong County in recent years. Since rainfall data during and after the September 2011 Sikkim earthquake was not available, we used the average daily rainfall (4.8 mm/day) from May 1, 2014, to July 31, 2017, as the input rainfall data for the purpose of validation.

## Supplementary information

Supplementary information.

## Data Availability

The relevant datasets of this study can be available from the corresponding author.
