# Supplementary material for: Failure probability assessment of landslides triggered by earthquakes and rainfall: a case study in Yadong County, Tibet, China
Source: Sci Rep. 2020 Oct 5;10:16531. doi: 10.1038/s41598-020-73727-4 (PMC7536195; doi:10.1038/s41598-020-73727-4)
Supplement: Supplementary file 1 — Supplementary information. [file 41598_2020_73727_MOESM1_ESM.pdf]

Supplementary Information for:

Failure probability assessment of landslides triggered by earthquakes and rainfall: A case study in Yadong County, Tibet, China

Lixia Chen[1*], Le Mei[1], Bin Zeng[2], Kunlong Yin[3], Dhruba Pikha Shrestha[4], Juan Du[5]

[1]Institute of Geophysics and Geomatics, China University of Geosciences, Wuhan, 430074, China

[2]Environment Faculty, China University of Geosciences, Wuhan, 430074, China

[3]Engineering Faculty, China University of Geosciences, Wuhan, 430074, China

[4]Department of Earth Systems Analysis, Faculty of Geo-Information Science and Earth Observation (ITC), University of Twente, Enschede, 7500 AE, the Netherlands

[5]Three Gorges Research Centre for geo-hazard, Ministry of Education, China University of Geosciences (Wuhan), Wuhan, 430074, China

[*]*Correspondence to:* Lixia Chen (**lixiachen@cug.edu.cn**)

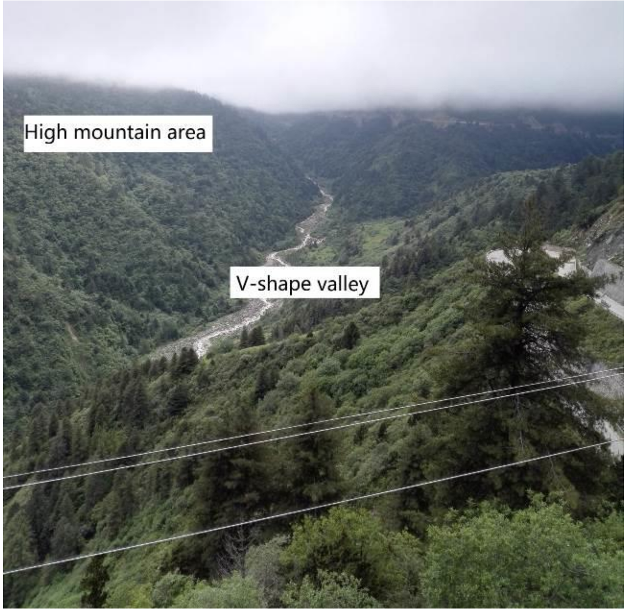

Fig. S1 Distant view of southern Yadong County. The Yadong river is visible in the V-shaped valley.

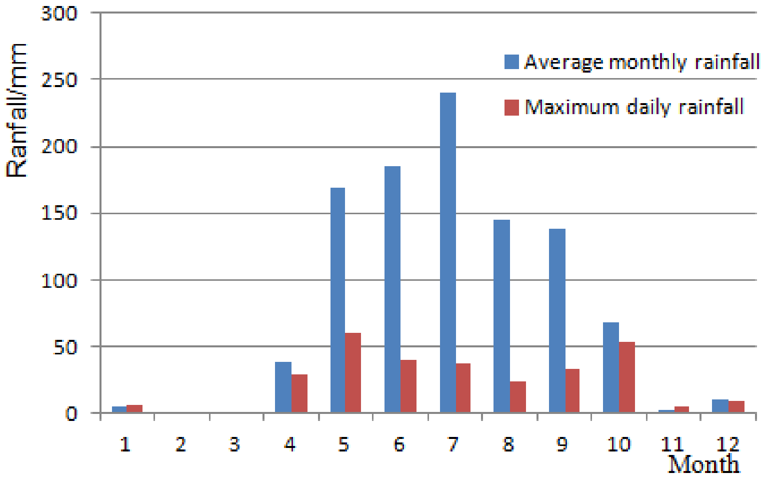

Fig. S2 Average monthly and maximum daily rainfall in the study area, based on rainfall data from 2014-2017.

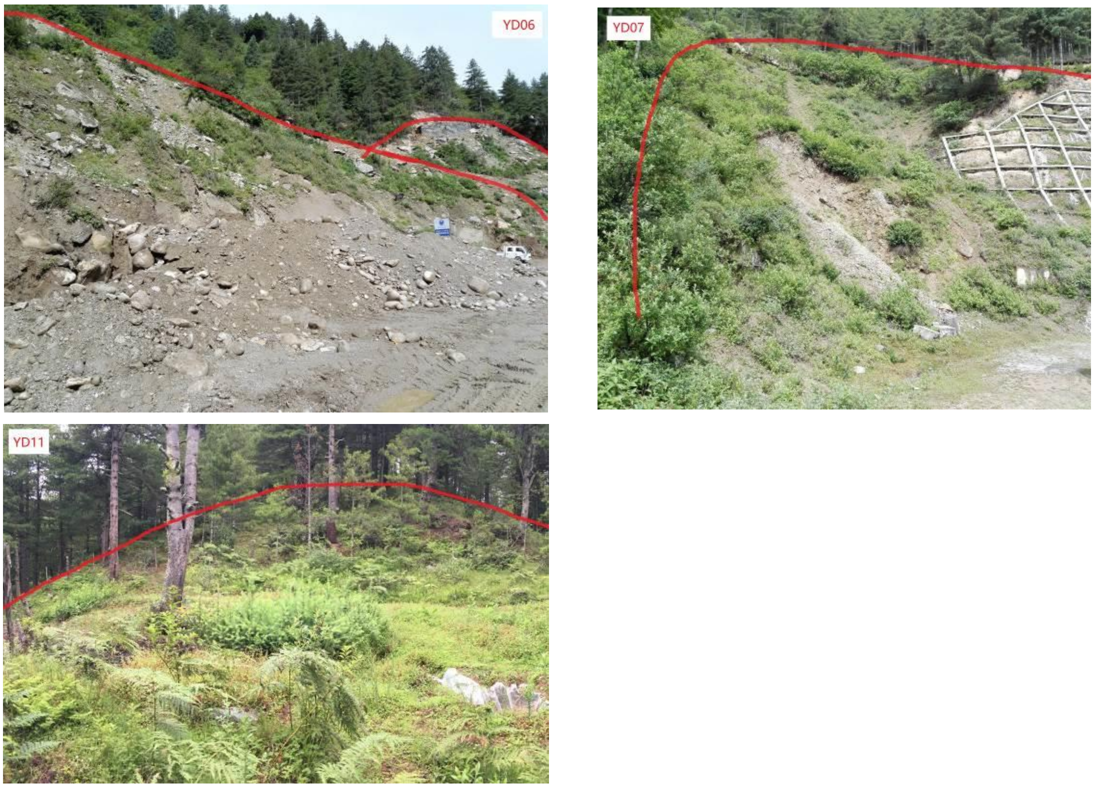

Fig. S3 Photographs of some debris slides induced by the Sikkim earthquake (YD06 and YD07) and by rainfall (YD11) in Yadong.

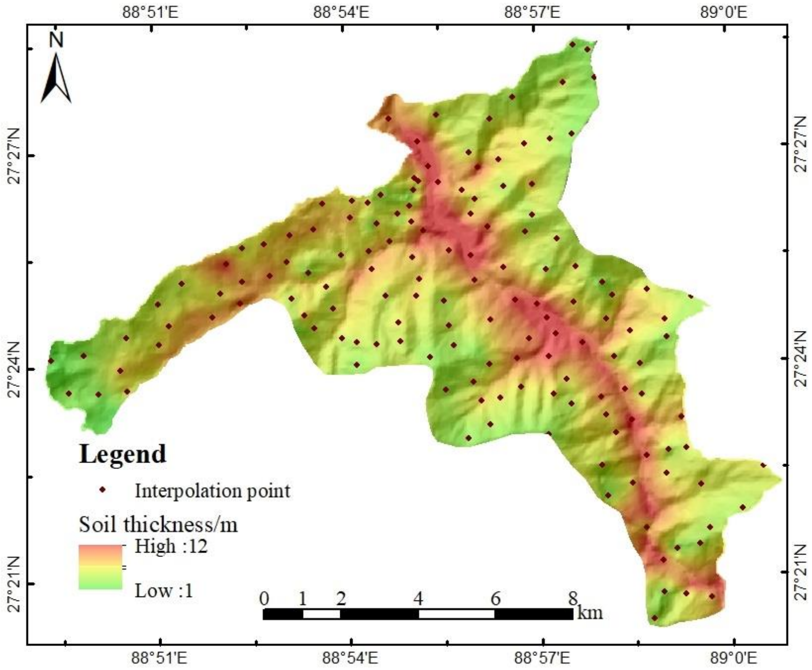

Fig. S4 Soil thickness map interpolated by ordinary kriging. This map generated by Le Mei

and Lixia Chen using ArcGIS ver. 10.2.

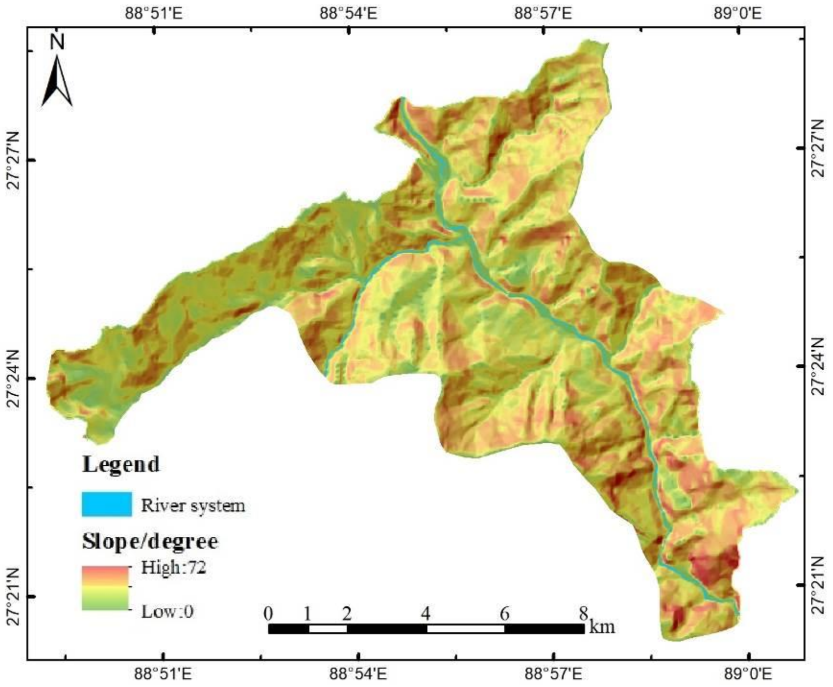

Fig. S5 Slope angle map of the study area. This map was generated by Le Mei and Lixia Chen using ArcGIS ver. 10.2. from a 25 m resolution DEM, downloaded from the National Catalogue Service for Geographic Information System (http://www.webmap.cn/main.do?method=index).

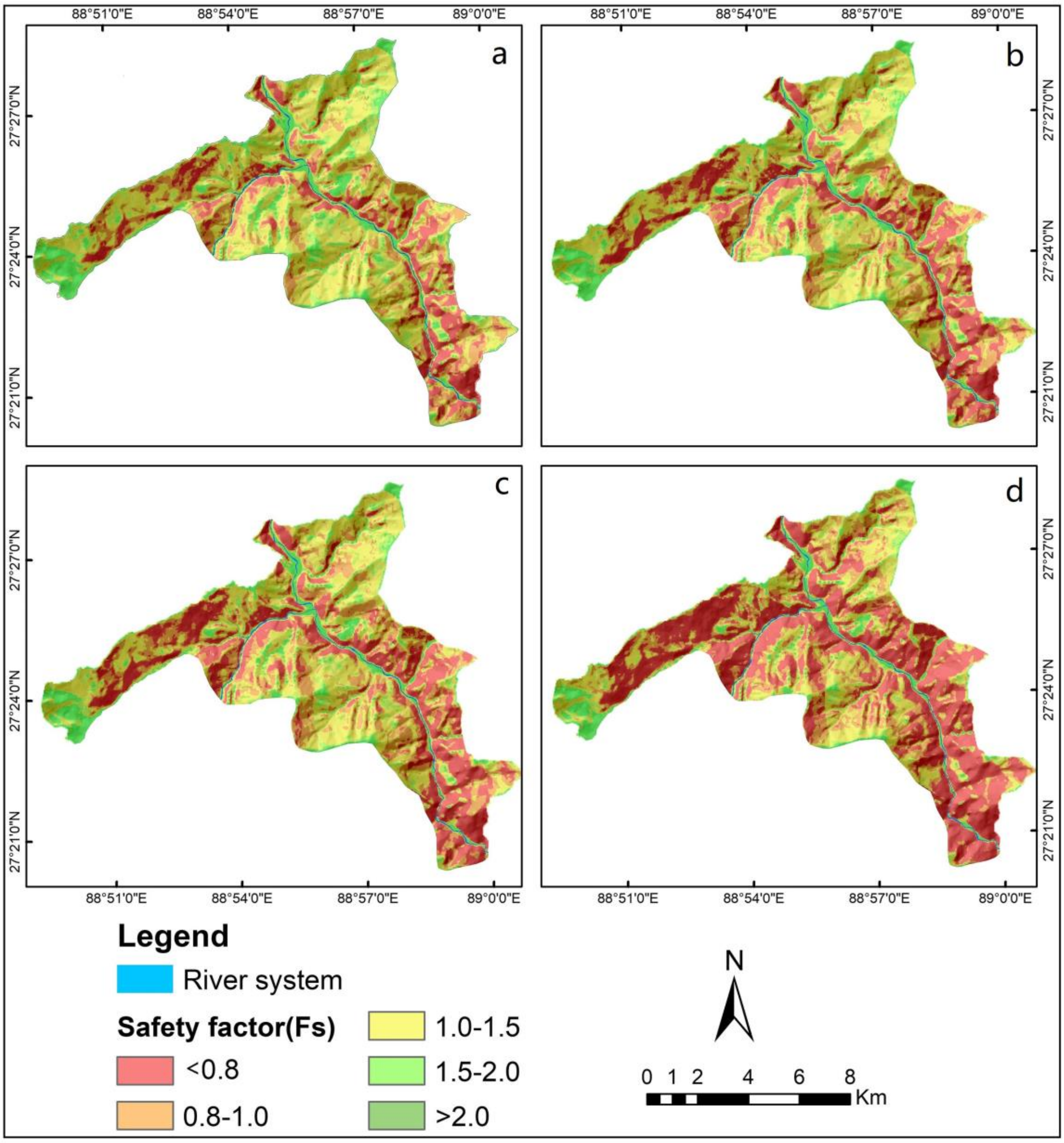

Fig. S6 Factor of safety maps in the study area under (A) dry conditions and rainfall of (B) 10 mm/d, (C) 20 mm/d, and (D) 45 mm/d. These maps were generated by Le Mei and Lixia Chen using Equation 2 using ArcGIS ver. 10.2.

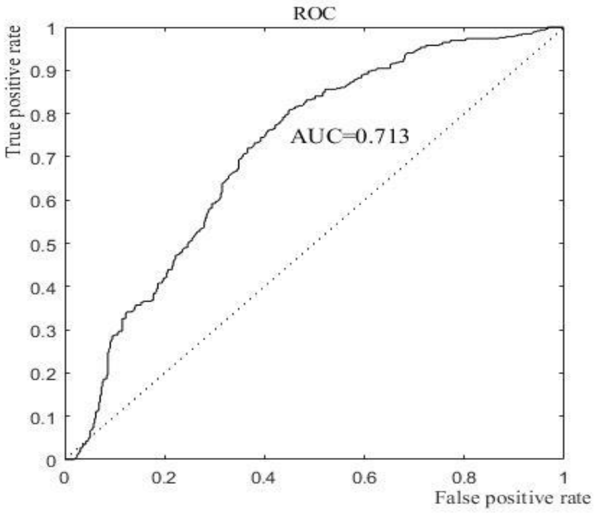

Fig. S7 Receiver operating characteristic curve to assess the accuracy of permanent displacement during the 2011 Sikkim earthquake.

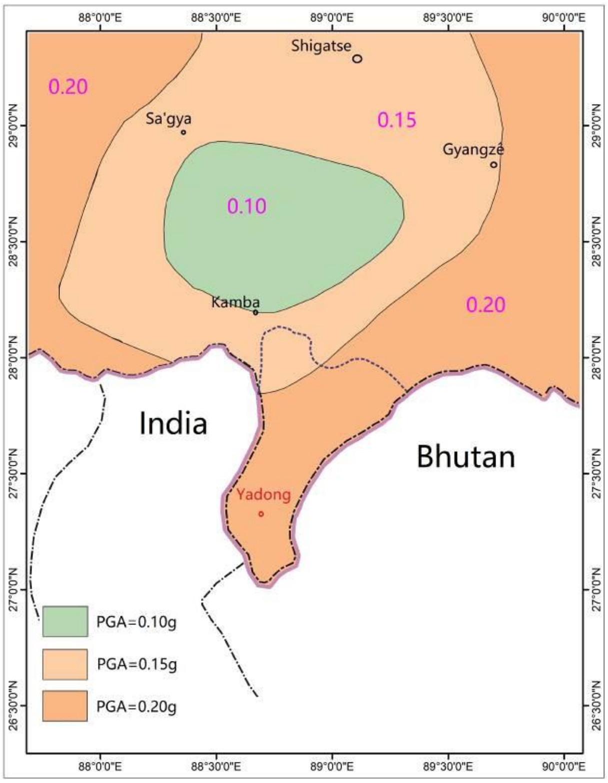

Fig. S8 Map of the 50-year 10% probability exceedance of peak ground acceleration values in Yadong and the surrounding area, downloaded from the Seismic Ground Motion Parameter Zonation Map of China (http://www.gb18306.cn/) by Le Mei and Lixia Chen.

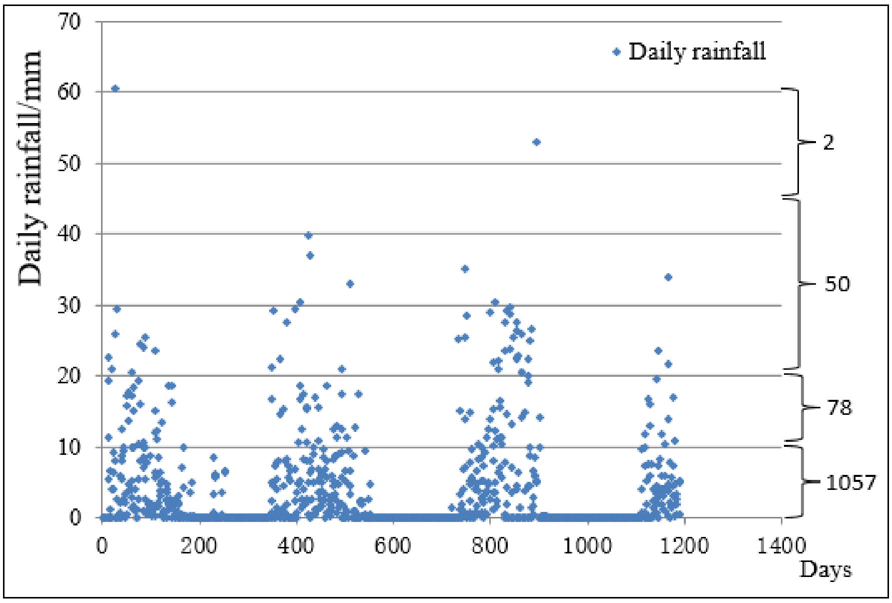

Fig. S9 Time series plot of daily precipitation totals in Yadong during the period 1 May 2014 to 31 July 2017. The four intervals used in this study (0–10 mm/d, 10–20 mm/d, 20–45 mm/d, and above 45 mm/d) are depicted, along with the number of days that fell within each interval.

Triggering events	Volume ($\times 10^4$ m^3)			Sum
	<1	1–10	10–100	
Sikkim earthquake(09/2011)	13	15	4	32
Rainfall(06/2014)	3	2	1	6
Gorkha earthquake(04/2015)	1	5	0	6
Unknown	5	1	0	6
Sum	22	23	5	50

Table S1. Inventory of landslides identified in Yadong for this study. Landslides are grouped by triggering event and by volume, with sums provided for each trigger. Landslide volume was estimated in the field by evaluating and calculating landslide length, width and depth.

ID	Area × 10000 m^2	Volume × 10000 m^3	Lithology	Material	Types	Triggering events
YD01	2.34	18.24	Gneiss	Soil	Debris slide	Sikkim earthquake
YD02	1.47	6.79	Gneiss	Soil	Debris slide	Sikkim earthquake
YD03	12.47	82.28	Gneiss	Rock and soil	Debris slide	Sikkim earthquake
YD04	9.22	77.27	Gneiss	Rock	Debris slide	Sikkim earthquake
YD05	7.82	44.36	Gneiss	Soil	Debris slide	Sikkim earthquake
YD06	0.06	0.25	Gneiss	Soil	Debris slide	Sikkim earthquake
YD07	0.02	0.19	Gneiss	Soil	Debris slide	Sikkim earthquake
YD08	0.99	3.99	Migmatite	Soil	Debris slide	Rainfall
YD09	1.40	6.90	Gneiss	Soil	Debris slide	Rainfall
YD10	1.56	16.20	Migmatite	Soil	Debris slide	Rainfall
YD11	0.18	1.84	Migmatite	Soil	Debris slide	Rainfall

Table S2. Detailed information about some of the landslides in the study area.

Daily rainfall/（mm d^{-1}）	Grids in the whole area	Area percentage	
		to the whole area	to rainfall-induced landslide
permanently unstable zone	8807	8.36%	0
0–10	15089	14.32%	21.62%
10–20	13977	13.27%	5.4%
20–45	11618	20.94%	37.84%
45–61	6376	6.05%	0
>61	38975	37.01%	35.14%

Table S3. Percentage of the study area covered by each rainfall threshold interval. The percentage of landslides triggered by each rainfall threshold is also shown.(Note: Grid size is 25 m × 25 m.)

D_N/cm	*P(f)*
0.05	0.023
1	0.061
2	0.087
3	0.084
4	0.128
6	0.147
9	0.186

Table S4. Permanent displacement, D_N, and landslide failure probability, *P(f)*, during the 2011 Sikkim earthquake.

Geologic unit	Dry condition			Saturated condition			permeability coefficient
	c /Kpa	φ /degree	γ /KN m^{-3}	c /Kpa	φ /degree	γ /KN m^{-3}	K/m s^{-1}
Granite	35	20	25	28	15	26	1×10^{-5}
Gneiss	20	15	20	16.5	11.5	21	6×10^{-6}
Migmatite	30	17	20	25.5	14	21	8×10^{-6}
c is the soil cohesion, φ is the friction angle, and γ is the specific weight							

Table S5. Geotechnical parameters of soil weathered from different bedrock types in the study area. Geotechnical parameters were not defined for the Quaternary deposit, as it is concentrated in a small area along both sides of the river, where the threat of landslide is low.
